# Rhinoplasty Education During Residency: Discussion of Current Barriers, Challenges, and Opportunities for Improvement

**DOI:** 10.1007/s00266-025-05539-8

**Published:** 2026-02-02

**Authors:** Sumun Khetpal, Anne E. Hall, Yasmine Ibrahim, Vishad Nabili, Michael R. Delong, Jason Roostaeian

**Affiliations:** 1 Division of Plastic and Reconstructive Surgery, University of California - Los Angeles, Los Angeles, CA USA; 2 Division of Facial Plastic Surgery, Department of Head and Neck Surgery, University of California - Los Angeles, Los Angeles, CA USA

**Keywords:** Rhinoplasty, Education, Plastic surgery residency

## Abstract

**Background:**

Rhinoplasty is a complex operation that warrants careful consideration of both functional and aesthetic principles. Despite its prevalence within plastic surgery and otolaryngology—head and neck surgery fields—its exposure and education are highly variable among training programs. The purpose of this study is to identify the various supplemental methods utilized outside of the operating room for educating residents on the technical and clinical aspects of rhinoplasty and evaluate the outcomes associated with various educational approaches.

**Methods:**

A scoping review was conducted using the PubMed/MEDLINE database using a combination of the following key terms: “rhinoplasty,” “rhinoplasty education,” and “resident training.” Articles were included that 1) discussed methods of educating residents on the technical and clinical aspects of rhinoplasty and 2) reported outcomes to objectively assess such methods.

**Results:**

Thirteen studies were included in the analysis. The majority of the studies discussed three-dimensional printed models and surgical simulators, followed by cadaver laboratories, detailed rhinoplasty educational programming, and video-assisted learning tools. In terms of assessed outcomes, surveys were utilized to assess residents’ confidence before and after intervention, test their knowledge on various clinical aspects, and their perceived effectiveness of these tools.

**Conclusion:**

This study highlights the various measures taken to provide education and instruction on rhinoplasty. Beyond intra-operating teaching and modeling, surgical simulators are highly effective and valuable for residents to practice technical maneuvers; however, future efforts leveraging artificial intelligence and software technologies can help further improve rhinoplasty education.

**Level of Evidence IV:**

This journal requires that authors assign a level of evidence to each article. For a full description of these Evidence-Based Medicine ratings, please refer to the Table of Contents or the online  Instructions to Authors  www.springer.com/00266.

## Introduction

Rhinoplasty is a complex operation that warrants careful consideration of both functional and aesthetic principles. According to The International Society of Aesthetic Plastic Surgery, over one million rhinoplasties were performed worldwide in 2023, making it one of the most commonly performed procedures in the fields of otolaryngology—head and neck surgery and plastic surgery [1].

Rhinoplasty is often considered among the most difficult operations to master, given its reliance on nuanced aesthetic judgment and multilayered anatomy. Considering the prevalence and complexity of rhinoplasty, it is imperative to discuss the key factors influencing the ability of resident trainees to learn and perform the procedure in their surgical practice. These factors include, but are not limited to, limited exposure to rhinoplasty during residency training, varying levels of personal interest in the procedure, unequal access to faculty mentors, differences in patient population (aesthetic versus reconstructive, primary versus revision), and varying opportunities to practice the technical skills and maneuvers associated with the procedure. Furthermore, the scope of surgical indications varies across institutions—academically inclined hospitals often performing more functional or cleft rhinoplasties whereas other programs address aesthetic concerns of patients. Similarly, faculty mentors often diverge in training (i.e., otolaryngology—head and neck surgery versus plastic surgery—aesthetic versus craniofacial fellowship), which will inevitably impact their approach to rhinoplasty and to resident involvement in the procedure. Additionally, in aesthetic and private practice settings, other constraints may limit trainee participation, as patients often expect direct attending involvement and may be less amenable to resident assistance. Ultimately, this inherent heterogeneity leads to varying exposure and intra-operative teaching to rhinoplasty, making it difficult to assess and compare training across residency programs.

Several studies have been conducted to better assess the quality of aesthetic surgery training. McNichols et al. conducted a survey of plastic surgery residents and program directors in 2017 and found that while there have been increases in dedicated aesthetic rotations at institutions, senior residents had relatively lower confidence in performing facial procedures compared to those of breast and body [2]. Furthermore, members of the Academic Aesthetic Surgery Roundtable (AASR) published a survey in 2021 that found having residents participate in faculty aesthetic clinics, aesthetic research, and discounted resident aesthetic surgery is strongly associated with the strength of an aesthetic surgery training programs [3].

While the literature has elucidated the rising prominence of aesthetic procedures in residency programs, clinical practice and instruction in rhinoplasty particularly presents challenges—namely due to its technical mastery and nuances surrounding aesthetic judgment. In a 2017 survey distributed to both residents and program directors within the American Society of Aesthetic Plastic Surgery, rhinoplasty was regarded as the most difficult operation to master [4]. Building on this, a systematic review by Zammit et al. revealed that simulation-based training is the most commonly employed method for rhinoplasty education (32%), followed by computer-based modeling (28%), cadaveric training (12%), and video-assisted methods (4%) [5]. However, despite the availability of these training modalities, significant gaps persist in perceived adequacy of exposure. Particularly, 72% of senior residents in the United States and Canada reported feeling adequately trained, compared to only 13% of junior residents [5]. Compounding this issue, confidence in performing rhinoplasty remains notably low, with the procedure receiving the highest number of "below satisfactory" confidence ratings across cosmetic procedures (*n*=116), while only twenty residents expressed "satisfactory" confidence [5].

Such observations highlight a gap in rhinoplasty education and present the opportunity to evaluate existing training methods and their effectiveness. Consequently, the purpose of this study is multi-fold: 1) to identify the various supplemental methods outside of the operating room utilized for educating residents on the technical and clinical aspects of rhinoplasty; 2) to evaluate the outcomes associated with these various educational approaches; 3) to identify barriers (if present) that may obstruct exposure and education of rhinoplasty; 4) to suggest alternative methods and solutions for residents to leverage for optimizing education and instruction within rhinoplasty. In performing this analysis, we hope to highlight the current state of exposure and education of rhinoplasty in residency training—and with this information, discuss future opportunities for enhancement among faculty members and societies within our field.

## Methods

**Fig. 1:**
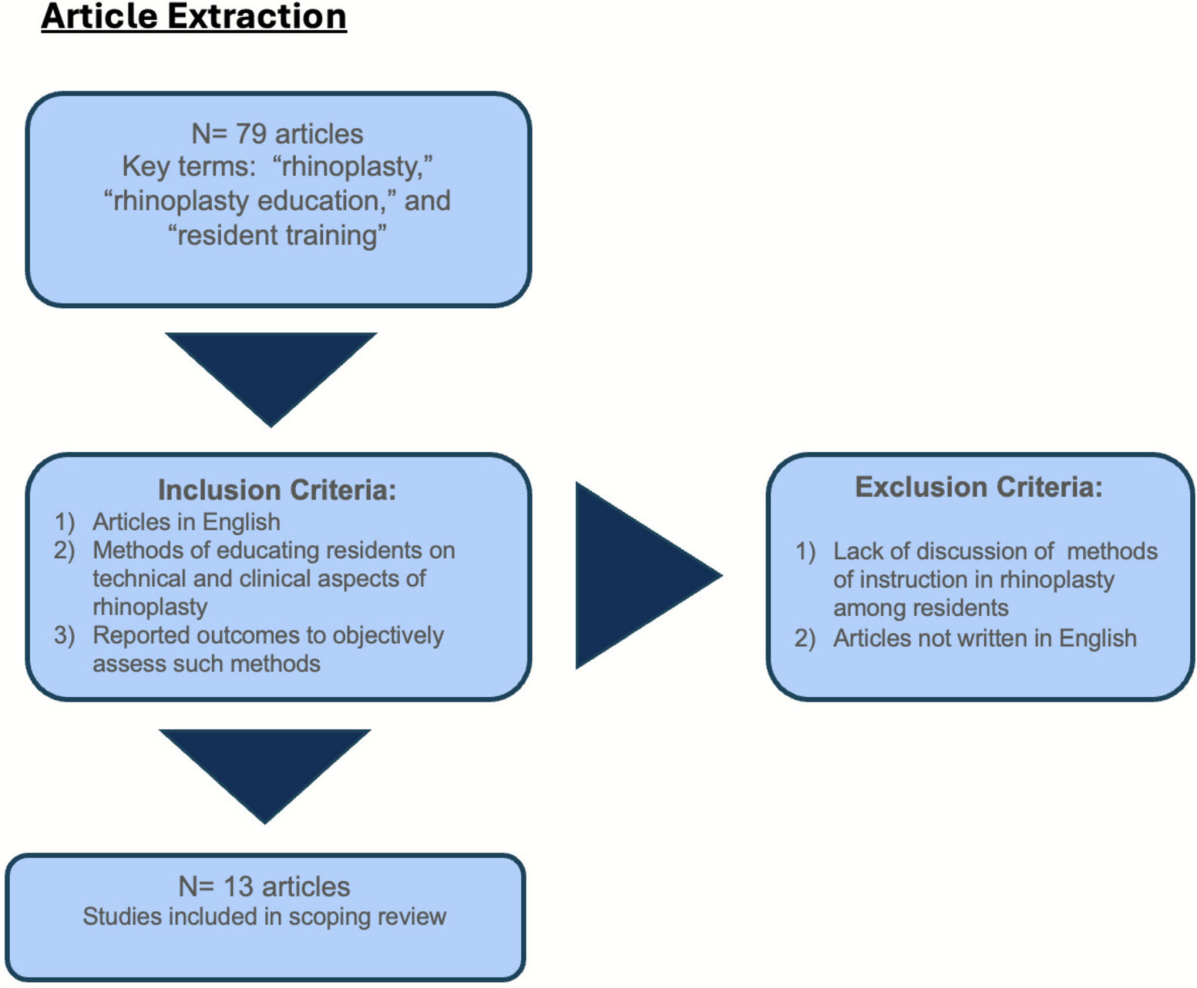
Article extraction

A scoping review was conducted using the PubMed/MEDLINE database using a combination of the following key terms: “rhinoplasty,” “rhinoplasty education,” and “resident training.” The review included articles that met the following criteria: 1) discussed supplemental methods of educating residents on the technical and clinical aspects of rhinoplasty; 2) reported outcomes to objectively assess such methods. Articles that were not published in English or did not provide a detailed description of the resident educational intervention were excluded. The process of article extraction can be visualized in Fig. 1. In order to reduce the possibility of bias, a standardized form was implemented for data collection; this was performed by S.K. and A.E.H. Variables collected included authors’ names, the year of publication, journal name, study design, sample size, educational method utilized, focused rhinoplasty technique, materials used, relevant outcomes of interest, and reported findings.

Educational methods were defined as surgical simulators (organic-animal, organic-cadaveric, inorganic-synthetic, inorganic-electronic) and specific rhinoplasty educational programming. Relevant reported outcomes included surveys assessing resident confidence, knowledge, and satisfaction, alongside performance metrics. Any conflicts were resolved through discussion and full-text review among the authors. The reference lists of all included studies were also hand-searched to identify any additional relevant articles. No funding was required to conduct this review of the literature.

## Results

A search of the PubMed/MEDLINE database resulted in a total of seventy-nine unique articles. After an abstract and full-text review, thirteen studies were included in the review (Table 1) [6–18]. Overall, seven of the studies derived from the plastic surgery literature, while the six came from the otolaryngology—head and neck surgery literature. The included studies, published between 2005 and 2024, explored a diverse array of rhinoplasty educational models. Among them, 69% (9/13) focused on surgical simulators (of that, 77.8% comprised of 3D-printed models, 22.2% of cadaver laboratories), while 23.0% (3/13) implemented detailed rhinoplasty educational programming, and 7.7% (1/13) introduced video-assisted learning tools.

**Table 1:** Rhinoplasty education intervention characteristics

Study	Journal of publication	Study design	Sample size	Educational method	Focused rhinoplasty technique	Materials used
AlReefi et al., [6]	Int forum allergy rhinol	Simulation	NA	3D septoplasty simulator	Septoplasty (endoscopic)	3D-printed models
Brandel et al., [7]	Ann plast surg	Case series (2012-2015)	34 rhinoplasty cases	Structured rhinoplasty educational program including patient education, consent, preoperative planning surgical techniques, structured postoperative management and follow-up. Also included supervision criteria for residents in the OR and clinical setting.	Holistic rhinoplasty surgical care and planning	NA
Chen et al., [8]	Otolaryngol head neck surg	Simulation	24 trainees	Hands-on cadaveric laboratory with 10-minute lecture on tools, landmarks, and objectives of lateral osteotomies	Lateral and percutaneous osteotomies	Cadaveric laboratories
Geelan-Hansen et al., [9]	Plast reconstr surg	RCT	20 trainees	Lecture on a systematic approach to nasal analysis focused on evaluating nasal photographs across frontal, lateral, oblique, and base views, with detailed criteria and the rule of 3’s to reinforce assessment.	Nasal analysis	10-slide powerpoint
Gupta et al., [10]	J Plast recon aesthet surg	Simulation	12 trainees	3D-printed model of sinonasal anatomy with an educational session	Sinonasal anatomy	3D-printed models
Jacovella et al., [11]	Aesthetic surgery journal	Simulation	25 trainees	40-hour cadaver-based training program based on nasal anatomy dissection and step-by-step development of rhinoplasty surgical skills	Performed rhinoplasty assisted by staff surgeon with skills including dorsum resection and lateral osteotomies	Cadaver laboratories
Oh et al., [12]	Laryngoscope	Simulation	22 (trainees=10; experts=12)	Surgical simulator	Spreader graft placement	Simulator with 3D-printed piece and porcine costal cartilages
Rahal et al., [13]	Otolaryngol head neck surg	Simulation	30 septoplasties	Video-assisted septoplasty	Septoplasty	Endoscope mounted on a nasal speculum
Robitschek et al., [14]	JAMA facial plast surg	Prospective observational (2015-2016)	20 trainees	Systematic approach model for nasal analysis	Nasal analysis	10-minute, 11-slide presentation
Schlegel et al., [15]	Otolaryngol head neck surg	Simulation	30 participants (19 attendings, 3 fellows, 5 trainees, and 3 medical students)	3D-printed nasal osteotomy task trainer with 20-minute lecture discussing relevant anatomy risks and benefits of techniques	Endonasal and percutaneous osteotomies	3D-printed models
Tumlin et al., [16]	Otolaryngol head neck surg	Prospective study	13 trainees	20-min lecture and nasal osteotomy simulator course (1.5 hours)	Osteotomies	3D-printed models
Wamkpah et al., [17]	PlB6ast surg global open	Simulation	37 trainees	Using 3D-printed models to help describe nasal anatomy	Nasal anatomy	3D-printed models
Zammit et al., [18]	Aesthetic surg J	Simulation	NA	A low-cost, step-specific simulator to help rhinoplasty teaching (specifically the 5 rhinoplasty learning areas of weakness)	1) nasal osteotomies; (2) caudal septum/anterior nasal spine manipulation; (3) nasal tip sutures; (4) cephalic trim; and (5) alar base modifications.	3D-printed models

This table summarizes key study and intervention characteristics, including author, year of publication, journal name, study design, sample size, educational method utilized, focused rhinoplasty technique(s), and materials used

*NA* not available, *RCT* randomized controlled trial

These methods primarily emphasized the technical aspects of rhinoplasty. Specifically, 30.8% (5/13) of studies focused on osteotomies, 23.0% (2/13) on septoplasties, and at least one study included specific rhinoplasty techniques including spreader graft placement, nasal tip sutures, cephalic trim, alar base modifications, and even a live rhinoplasty. Beyond technical practice, studies also explored the clinical aspects of rhinoplasty care, with 15.4% (4/13) on detailed sinonasal anatomy, while another 15.4% (2/13) emphasized nasal analysis, providing residents with a structured framework for learning and formally evaluating their mastery of the curriculum. Additionally, 7.7% (1/13) focused on holistic rhinoplasty surgical care and planning, addressing key topics such as patient education, informed consent, surgical planning and postoperative management.

In terms of resident educational outcomes, the majority of the studies evaluated model effectiveness (5/13), resident experience (including satisfaction and recommendations) (5/13), confidence (5/13), and knowledge (5/13). For the five studies that reported on model effectiveness, including usefulness, value, and realism, all studies demonstrated positive outcomes, emphasizing high face validity, educational benefits, and strong ratings for anatomical accuracy and realism [6, 12, 13, 16, 17]. As for the five articles that reported on resident experience including their satisfaction and likelihood of recommending the model to a colleague, residents expressed high levels of satisfaction, with strong support for integrating models into the curriculum and high likelihood of recommending their sessions to others [6, 7, 15–17]. Of the five studies that reported resident confidence before and after an educational intervention, all but one study reported increased resident confidence [7, 8, 15–17]. Particularly, there was a significant increase in confidence following cadaveric laboratory (*p*<0.0001) [8] and a simulator study focused on osteotomies, with improvements observed after the pre-simulator lecture (*p*<0.05) and additional gains following the simulator session (*p*<0.05) [16]. Similarly, all five studies that assessed changes in resident knowledge (three utilizing simulators and two deploying lectures) reported increased knowledge about the specific rhinoplasty technique following the intervention [9, 10, 14, 15, 17].

Of the three studies that reported on performance measures, Chen et al. demonstrated that residents achieved high completion rates of bony cuts for intranasal (96%) and percutaneous (75%) osteotomies (*p*=0.97) during the cadaver osteotomy laboratory simulation, highlighting not only their technical proficiency but also the effectiveness of cadaveric laboratories as a hands-on learning experience [8]. Additionally, Oh et al. found that when comparing the hand motion metrics when using a spreader graft placement simulator such as—total hand displacement, cumulative hand motion directions, and time to complete a suture task—attending surgeons significantly outperformed residents [12]. Lastly, Jacovella et al. found that residents who underwent the 40-hour cadaver rhinoplasty training performed better on their live rhinoplasty than those who did not, consistent with the study group’s higher “very good” scores (76% vs. 4%, *p*<0.001) [11].

Lastly, of the seven studies that developed and assessed 3D-printed models, 71.4% of studies reported on cost and 57.1% reported on production. For cost, four out of five models ranged from $2.15 to $132.60, with Wampkpah et al. being the most expensive at $550 [6, 15–18]. Production times for three models ranged from 15 minutes to 16 hours, with Zammit et al. requiring the longest at 20 hours per unit [6, 16–18]. A summary of findings can be seen in Table 2.

**Table 2:** Summary of findings

Study	Outcome	Metric	Finding
AlReefi et al., [6]	Model realism	Post-simulation questionnaire (5-point Likert Scale)	Average scores 4.05 ± 0.82 (anatomically correct) and 4.2 ± 1 (realistic).
	Resident experience	Post-simulation questionnaire (open-ended questions)	92% of residents desired the simulator to be integrated into their teaching curriculum.
	Time taken to perform septoplasty	Simulations were recorded by 2 independent raters	There was a significant difference (*p* < 0.05) between the expert, intermediate, and novice groups in time taken and nares cuts.
	Cost and production	Cost and production analysis	Final model was 186 CAD, and production time was 6 hours.
Brandel et al., [7]	Complication rates	Chart review	Complications included: asymmetry (10.5%), septal perforation (2.6%), and difficulty breathing (15.8%). Complication rate requiring revision in OR was 0%.
	Patient satisfaction	5-point Likert Scale questions on 6 satisfaction questions	Average patient satisfaction was rated 4.55/5.0 (SD); median response for all 6 satisfaction questions was 5/5 with only 1 respondent expressing dissatisfaction of any metric.
	Resident experience and confidence	5-point Likert Scale question on 6 satisfaction questions with the resident aesthetic clinic and confidence in aesthetic procedures	Residents reported a good autonomous experience (*p* < 0.001) and 90% reported confidence with rhinoplasty.
Chen et al., [8]	Resident confidence	5-point Likert Scale	Trainees reported increase confidence after cadaveric laboratory (*p* < 0.0001)
	Completion of bony cut and osteotomy placement	Evaluated for completeness of bony cuts and medial periosteal disruption as all-or-none phenomena. Osteotomy position was graded as correct or incorrect.	Bony cut was similar between intranasal and percutaneous osteotomies (*p* = 0.097). Completion of the bony cut was similar between intranasal and percutaneous osteotomies (96% vs 75%, *P* =. 097), as was correct placement of the osteotomy (75% vs 67%, *P* =. 530). Intranasal osteotomies were more likely to cause periosteal disruption (*p* = 0.020).
Gupta et al., [10]	Resident knowledge	18-point binary scale evaluating nasal analysis	Study group had overall higher scores in both initial assessment (p < 0.001) and a trend toward higher scores in follow-up assessment (0.069). Residents in the study group scored higher with: vertical nasal proportion (p < 0.009) and Fitzpatrick score (*p* = 0.003) in both initial and re-assessment.
	Resident knowledge	7-questionnaire survey on 5-point Likert Scale addressing familiarity with the goals of rhinoplasty and different rhinoplasty techniques	Residents demonstrated improvement in knowledge across all categories assessed, with the greatest improvement in knowledge in the categories they scored lowest in,
Jacovella et al., [11]	Performance metrics (rhinoplasty accuracy and number of attempts)	4-point rating scale	The study group demonstrated significantly better performance than the control group, with a higher proportion of "very good" scores (76% vs. 4%) and fewer "acceptable" or lower scores (*p*<0.001).
Oh et al., [12]	Time required to complete the suture task	Electromagnetic position sensing device	Experts performed the task in significantly less time (mean 254 s) than the residents (mean 312 s) (*p* < 0.05).
	Total hand displacement	Electromagnetic position sensing device	Experts had significantly less path length traveled for both “suture” task (4206.2mm experts, 6708.6mm residents,* p* < 0.001) and the “tie” task (2004.3mm, 2958.3mm residents,* p* < 0.01).
	Cumulative number of hand motion direction changes	Electromagnetic position sensing device	Experts had significantly less hand movements made for both “suture” task (192.6 experts, 334.8 residents, p < 0.001) and the “tie” task (108.6 experts, 195.4 residents,* p* < 0.001).
	Accuracy of suture insertion	Measured the distance from the suture insertion site to the target mark using ruler-referenced photographs analyzed with image processing software	Difference was not significant between the two groups.
	Subjects rating of validity and usefulness of training model	Questionnaire	Both residents and experts agreed on the face validity and usefulness of the trainer.
Rahal et al., [13]	Feasibility	Qualitative feedback	The primary surgeon considers the device to be user-friendly, with less than 5 min of set up at the beginning of each case and has not resulted in any significant increase in total operating time.
	Model usefulness	Qualitative feedback	Model allows for real-time visualization of all the steps of surgery on the video monitor in real time. Also, the mentor can guide the resident through the surgery and provide more appropriate, immediate feedback. All steps can be recorded for later educational use.
	Complications	Chart review	No reported complications with the technique.
Robitschek et al., [14]	Resident knowledge	Binary 18-point system evaluating nasal analysis	Study group demonstrated immediate improvement in nasal analysis score after lecture (*p*<0.001). At follow-up at 10 weeks, the study group had higher nasal analysis scores than control (*p*<0.001).
Schlegel et al., [15]	Resident confidence	5-point Likert scale	Increase in participant confidence across many categories.
	Resident knowledge	8-Question educational osteotomy quiz	Average score increased from 57.9% to 79.1% between pre- and post-simulation assessments.
	Resident experience	5-point Likert scale	Participants either agreed or strongly agreed that they benefited from the session (92.8%) and they would recommend this session to a peer (86%) and that the model was high fidelity (64%).
	Cost	Cost-analysis	$12.31 for one simulation set.
Tumlin et al., [16]	Cost and production time	Cost and production analysis	$2.15 and 15 minutes of production time per midface model.
	Resident confidence	5-point Likert scale	Residents experienced an increase in confidence from baseline to post-lecture and also post-lecture to post-simulation (*p* < 0.05 for both).
	Model value and resident recommendation	5-point Likert scale of 5-value assessment questions	Overall average value was 4.75/5 (0.45); with residents rating their likelihood to recommend the model to a colleague at 4.75/5 (0.45).
Wamkpah et al., [17]	Resident knowledge	Matching patient photos to models and describing relative anatomy	The median number of correctly matched model-photograph pairs was four (of six) and the median number of correctly labeled nasal analysis items was 15 (of 30). Senior residents had a statistically significantly better performance than junior residents in identifying correct model-photograph pairs (*p*<0.001) but not in labeling correct relationships of nasal bony and cartilaginous structures (*p*=0.176).
	Resident confidence on assessing nasal “anatomy” “function” “aesthetic” and “etiology”	VAS 0–100	The median differences between pre- and post-exercise self-ratings were not statistically significant in any milestone category.
	Model usefulness	VAS 0–100	The residents found the exercise to be useful (median VAS rating 85 of 100) and were highly likely to recommend its use in future (median VAS rating 87 of 100).
	Cost and production	Cost and production analysis	The model was estimated to cost approximately $550 per unit and require 12 to 16 hours to construct.
Zammit et al., [18]	Cost and production time	Cost and production analysis	The model was estimated to cost approximately 75 CAD per unit and 20 hours to construct.

This table outlines the major outcomes for each study, the metric employed, and the overall findings

*CAD* canadian dollar, *VAS* visual analogue scale

## Discussion

Simulation-driven learning has been a longstanding method of instruction within surgery for many years. Furthermore, in a study by Cardoso et al., the authors discuss three key principles of simulation training: experiential learning (providing trainees with immersive experiences in which they may practice skills and face consequences of their actions), cognitive load theory (emphasizing how learners process information and manage the mental load during learning), and constructivism (integrating new insights to existing mental models) [19]. Previously, a systematic review by Gill et al. found that rhinoplasty simulators, ranging from cadaveric and live animal models to virtual and 3D-printed systems, have demonstrated significant benefits in enhancing trainee confidence and knowledge, though the current literature primarily focuses on the development of simulators rather their validation and educational utility [20]. This study has outlined the multiple types of simulation to facilitate education and exposure to rhinoplasty, while synthesizing the reported outcomes with such methods. However, it is important to note these are static models that have several limitations that may ultimately impact the ability of residents to perform rhinoplasty in clinical practice. These include the following: 1) no real-time feedback from faculty attendings, 2) cost of such technologies and equipment and therefore, challenges in sustainability; 3) difficulty in representing true surgical conditions; 4) difficulty in reflecting variabilities of preoperative nasal anatomy and encapsulating multiple types of rhinoplasty (i.e., cleft, cosmetic, trauma); 5) difficulty in replicating different media of tissue (i.e., cartilage, bone) within models [19] (Fig. 1).

### Barriers to Learning Rhinoplasty—A Resident’s Perspective

**Fig. 2:**
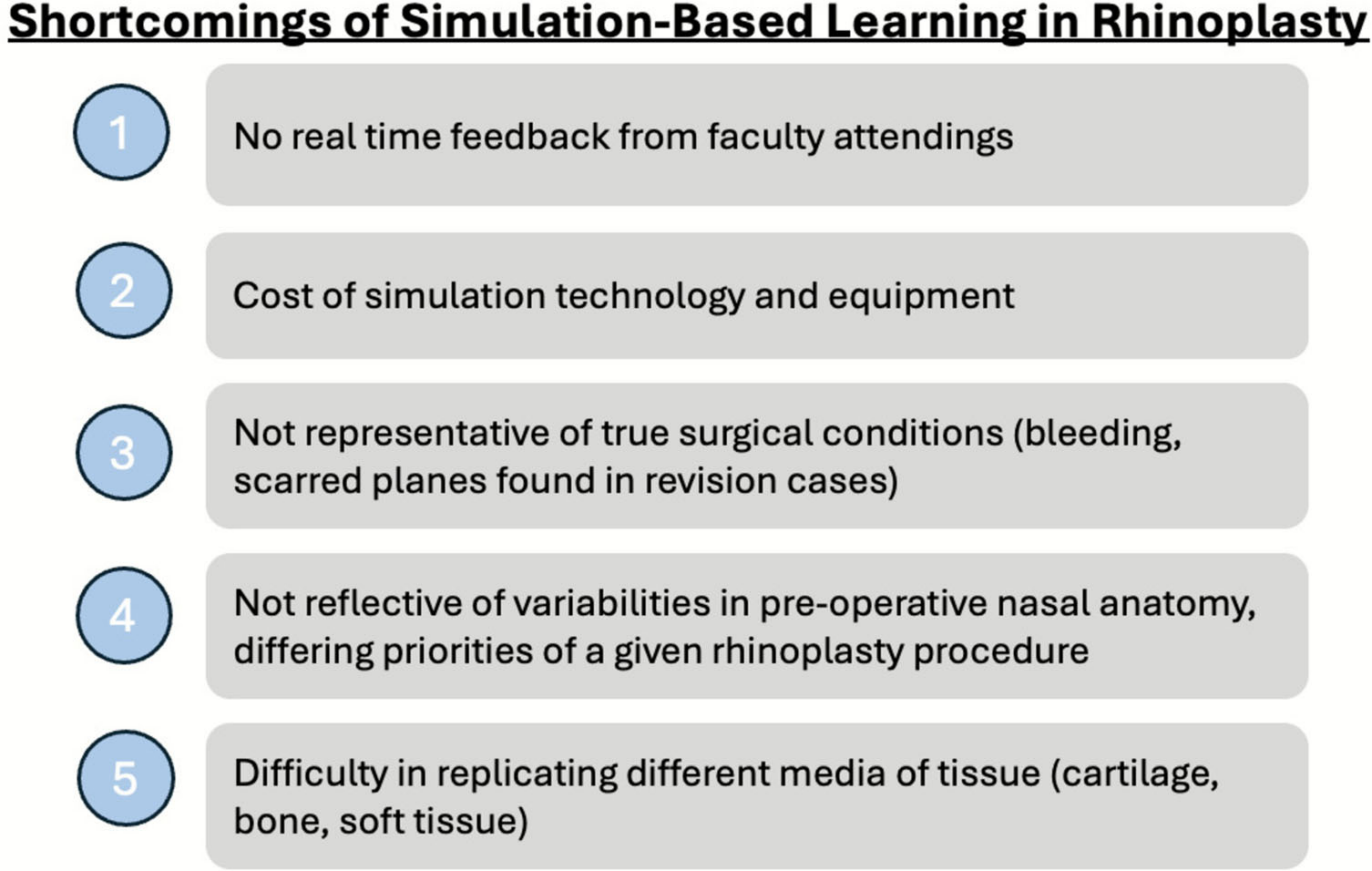
Shortcomings of simulation-based learning in rhinoplasty

**Fig. 3:**
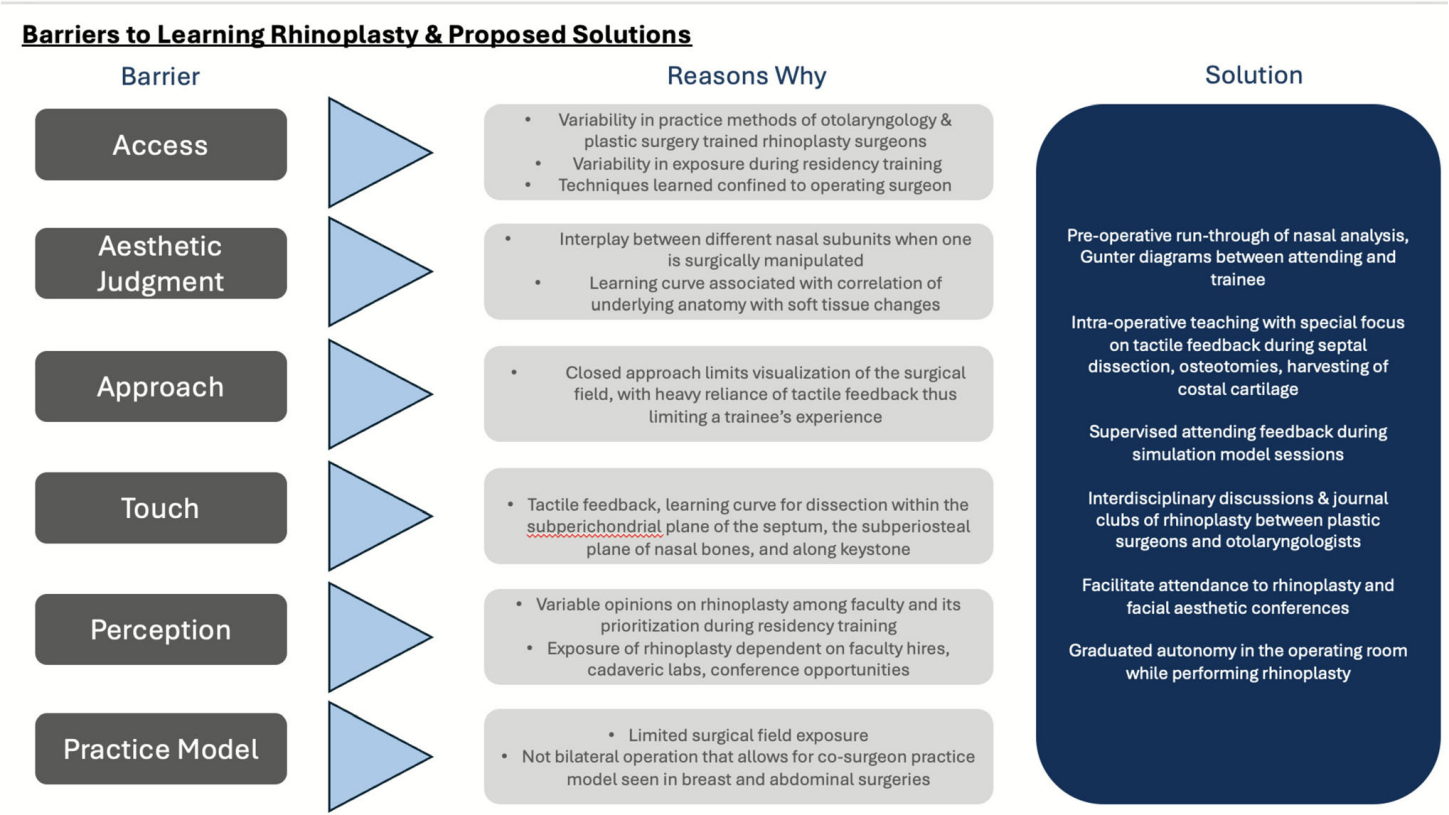
Barriers to learning rhinoplasty and proposed solutions

Given its aesthetic and functional priorities, rhinoplasty is often regarded as one of the most complex operations within plastic surgery and otolaryngology—head and neck surgery. There are multiple reasons that account for the difficulty of the procedure, and we find that many of these become apparent and compound during residency Fig. 2. Furthermore, these barriers may ultimately impact how trainees approach their clinical practice and their inclusion (or lack thereof) of rhinoplasty. We have included several of these considerations below. These are summarized in Fig. 3.

#### Access

As mentioned previously, resident exposure to rhinoplasty is highly dependent on institutional factors, including the availability of cosmetic faculty and case volume. Programs with limited aesthetic mentorship or fewer cosmetic cases may offer fewer operative opportunities, which can significantly impact resident confidence and skill development. Additionally, within their respective institutions, plastic surgery and otolaryngology—head and neck surgery residents—may face dramatic differences within their mere exposure to rhinoplasty during training. Thus, depending on their designated specialty, specific residents may or may not have consistent exposure to the procedure. In addition, the extent of their clinical exposure is confined to the practice scope of the attending surgeon. Whether the attending performs open or closed technique, whether they utilize an endoscope for addressing nasal airway obstructive symptoms, whether they practice dorsal preservation, whether they care for patients with aesthetic concerns versus those with cleft deformities, these have massive implications for residents and their collective exposure to rhinoplasty.

#### Aesthetic Judgment

Perhaps, one of the most challenging aspects of rhinoplasty is not only deliberate and accurate nasal analysis, but also the synthesis of preoperative nasal anatomy with the necessary surgical maneuvers to achieve a given patient’s desired functional and aesthetic result. Imaging softwares including Vectra have often been utilized during preoperative consultations to provide simulations of postoperative results to patients. This discussion may entail high-level mention of particular maneuvers to the patient; for trainees, this is high variability in the extent of preoperative discussion and surgical planning that occurs with attending surgeons. Much of education is confined to the operating room. To enhance this opportunity, we find that trainees and attending surgeons should have preemptive discussion of the patient’s preoperative anatomy, perform comprehensive nasal analysis, and propose particular surgical maneuvers given the aforementioned information. Throughout the procedure, the attending surgeon should verbalize particularly important steps relevant to their decision making; trainees should remain inquisitive while they actively assist. Artificial intelligence technologies, such as Hololens, could facilitate the interplay between surgical manipulation of nasal anatomy and the resultant soft tissue aesthetic.

#### Approach

Technically speaking, rhinoplasty can be performed through either open, closed, or hybrid approaches. Surgical field visualization is ideal during open approaches, as the trainee can actively assist and appreciate not only how particular technical maneuvers are performed, but also realize their collective impact on the overall nasal aesthetic. A closed approach may be performed during functional cases, during which septoplasty and turbinate reduction are performed. A nasal endoscope may facilitate visualization and learning, but spatial anatomic relationships should be clarified intra-operative to enhance learning. One can argue that given the prevalence of nasal endoscopy within otolaryngology—head neck surgery, plastic surgery—residency programs should recruit this expertise as an additional adjunct to rhinoplasty education.

#### Touch

In much of surgery, the reliance on tactile feedback cannot be understated. While surgical simulations, namely cadaveric and animal dissections, are helpful among trainees, attending surgeons should prioritize learning of particular maneuvers that rely on appropriate tactile feedback for success [21]. These include but are not limited to access to the subperichondrial plane of the septum, the subperiosteal plane of the nasal bones, and the transition of the upper lateral cartilages to the nasal bones near the keystone, are particularly important to appreciate during rhinoplasty.

#### Perception

Within academic residency programs, the program leadership and faculty members may have variable opinions surrounding instruction within rhinoplasty among trainees. Furthermore, while the ACGME does regard the procedure as a fundamental procedure within their respective fields, others may regard rhinoplasty as largely cosmetic and thus may de-emphasize its importance during training. As a result, the degree of exposure to rhinoplasty is largely dependent on how the training program prioritizes the procedure—whether through hiring of faculty that perform rhinoplasty, encouraging cadaver or simulation labs on facial aesthetics, or enabling residents to attend rhinoplasty symposium during training.

#### Practice Model

Within plastic surgery residency, many of the procedures (namely abdomen or breast) are amenable to a co-surgeon model due to their inherent nature of being bilateral. Moreover, during a breast reduction or gender-affirming mastectomy, an attending surgeon often allows their resident to effectively “own” their side. While the resident’s confidence and competency will vary by post-graduate year, there is a sense of graduated autonomy and development of rapport with their attending over time. Furthermore, such procedures also offer a larger visual field that allows for direct, constructive feedback given that the attending surgeon and trainee can both appreciate the surgical steps in synchrony. On the other hand, rhinoplasty is an operation in which experimental learning and attending-driven feedback are often difficult. This often results in a high proportion of rhinoplasty exposure that is observational as opposed to hands-on acquisition of surgical maneuvers in the operating room.

### Future Directions

**Fig. 4:**
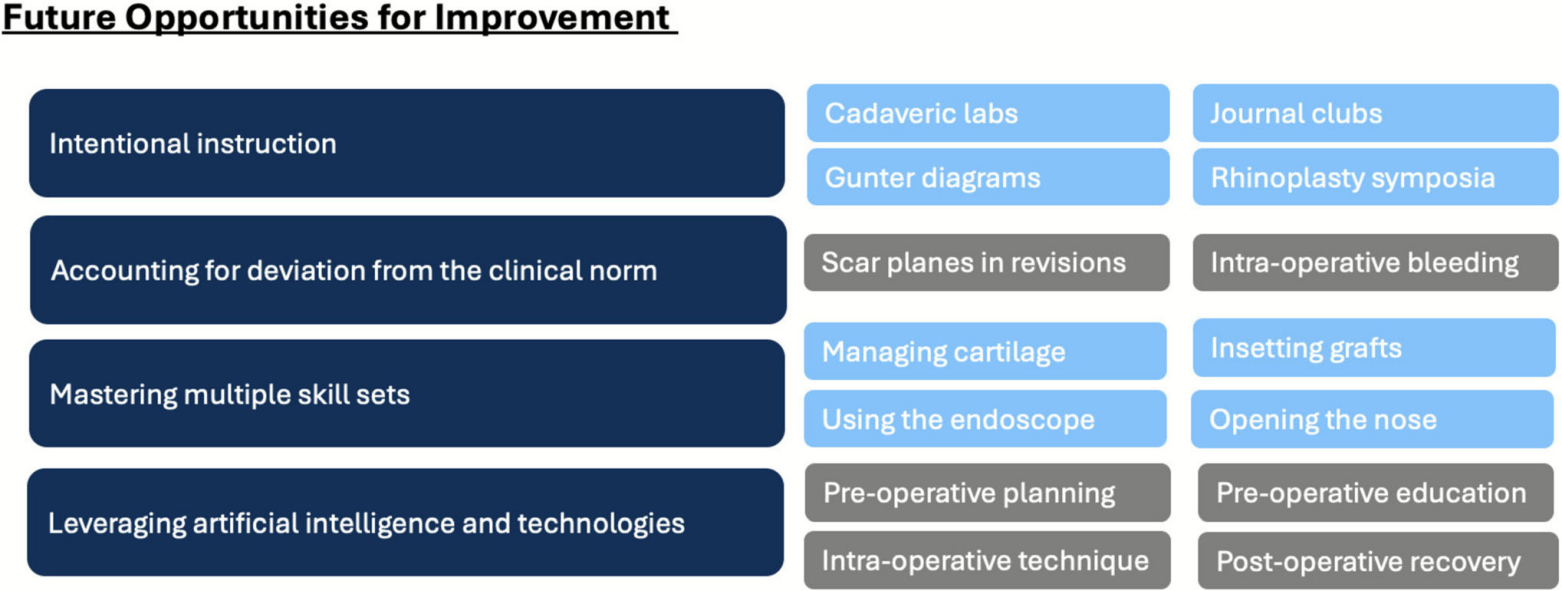
Future opportunities for improvement

To account for the above barriers and the progress made in surgical simulation, we would like to propose several considerations to enhance the learning environment for residents interested in rhinoplasty. This is summarized in Fig. 4.

#### Intentional Instruction

In rhinoplasty, we find that certain maneuvers—namely septal dissection and osteotomies—require specialized training for residents [22]. Particularly, simulation models and training sessions should not only assist in the visualization of anatomy, but also provide opportunities for constructive feedback and evaluation. This can be done with direct observation by attendings or by formal review of surgical videos. Moreover, the distinction between passive and active instruction cannot be understated. Intentional feedback and instruction allow for continual revision of techniques for improvement of techniques and ultimate aesthetic results. Beyond these simulated experiences, a similar learning mindset should be embodied in the operating room; the attending surgeon should verbalize maneuvers as well as particular notable decision making points during the operation. Preoperative comprehensive nasal analysis between attending and trainee should be performed. Gunter diagrams may be utilized for strategy and planning of certain cartilage grafts and their respective functions within the nose. These activities will further engage the trainee as they become more acquainted and immersed within rhinoplasty. Beyond the operating room, rhinoplasty educational opportunities—whether didactic lectures, journal clubs, cadaveric labs, and conferences—should be offered to residents.

#### Accounting for Deviation from the Clinical Norm

Amidst their multiple advantages, surgical simulations do not represent the real clinical conditions within the operating room. Particularly within rhinoplasty, appropriate visualization of surgical field may be often confounded by intra-operative bleeding; as a result, access to subperichondrial and subperiosteal planes may be more challenging compared to that in a cadaver or animal model. Furthermore, revision rhinoplasties are also subject to the distortion of planes and may challenge residents when first performing rhinoplasty. While stationary surgical simulations have served as valuable, they could be further improved and revised to depict these real-life conditions of rhinoplasty [23]. One may also consider the cost implications associated with reflecting such nuances within learning models. There may be opportunities instead to leverage software technologies to instead instruct residents on how to mitigate these challenges during the procedure.

#### Mastering Multiple Skill Sets

Rhinoplasty involves mastery of multiple skills including but not limited to cartilage harvest, cartilage carving, placement of grafts, and management of the nasal airway. Furthermore, each rhinoplasty presents a variable set of priorities for a variety of applications. For instance, some patients may be undergoing rhinoplasty for purpose of exclusively addressing nasal airway obstructive symptoms; therefore, maneuvers such as turbinate outfracture, submucosal reduction of turbinates, and septoplasty, are paramount to learn and practice for the assurance of nasal airways patency. Other rhinoplasties may involve particular focus toward the dorsum—i.e., the relationship among the nasal bones, upper lateral cartilages, and cartilaginous septum. Attention may be directed toward the tip complex and specifically, how the lateral crura sit in relation to the domes. Beyond focusing on the different nasal subunits and their respective relationships, the act of rationing cartilage for various grafts (i.e., spreader, caudal septal extension graft) is an important skill among trainees; the act of carving cartilage and being cognizant of warping tendencies is paramount in designing and placing grafts. The act of harvesting cartilage—whether a septal L-strut or autologous costal—assumes a learning curve among trainees. Furthermore, faculty attendings should approach instruction through a focused mindset of acquired skills and integrate their instruction during rhinoplasty.

#### Leveraging Artificial Intelligence and Technology Solutions

During residency, trainees often resort to learning by active observation and modeling in rhinoplasty. There is an understated potential and opportunity to integrate artificial intelligence and other technologies to demystify and advance education in rhinoplasty. Technologies, such as Google Glass and Microsoft HoloLens, can be leveraged as well intra-operatively—thus allowing for residents to capture and record these important steps during the procedure. Software technologies may be built to correlate certain techniques and anatomic configurations with three-dimensional contours for the trainee. In addition, technologies such as Vectra can facilitate teaching of aesthetic goals and complement discussions of anticipated technical maneuvers and manipulation of the underlying anatomy. In addition to fixating on the technical aspects of rhinoplasty, artificial intelligence solutions have also been vital in guiding initial preoperative consultations as well as postoperative recovery period among patients; this can ultimately help residents who are navigating the entire care journey among patients undergoing rhinoplasty.

### Limitations

There are a number of limitations that warrant consideration. First, this study consists of articles that showcase a single intervention—whether a cadaveric dissection or surgical software simulation—but do not provide longitudinal outcomes on how the experience specifically impacted resident education. Further studies could assess how these correlated with a given resident’s confidence or aptitude in performing rhinoplasty. Second, many of these studies did not provide information on the cost of the analyzed resource; moreover, the sustainability and ultimate impact of these resources may be questioned if prices are exorbitant for programs to incur. Third, this study did not elicit particular opinions of residents regarding their experiences in practicing and mastering rhinoplasty. Future studies could survey residents—both from plastic surgery and otolaryngology—head and neck surgery—to better understand their sentiments and confidence toward specific technical maneuvers within rhinoplasty. It would be interesting to understand how cognitive load associated with these simulations correlated to performance measures. Additionally, attending surgeons could be asked about how they approach teaching rhinoplasty, and how these processes may have evolved over time. Fourth, this analysis explicitly focused on the technical aspects of rhinoplasty and in doing so, did not discuss how aesthetic and clinical judgment are approached during rhinoplasty instruction. Further efforts by plastic surgery and otolaryngology—head and neck surgery residency programs—can be made to ultimately enhance this aspect of education within rhinoplasty.

## Conclusion

This study highlights the various measures taken to provide education and instruction on rhinoplasty. Beyond intra-operating teaching and modeling, surgical simulators are highly effective and valuable for residents to practice technical maneuvers including septal dissection and osteotomies. Despite their prevalence, there remain barriers toward learning rhinoplasty including but not limited to access to the procedure across otolaryngology—head and neck surgery and plastic surgery—mastery of aesthetic judgment, surgical approach, reliance on tactile feedback, perception of the procedure in academic plastic surgery, and practice model for resident education [24–26]. Future studies could explore how aesthetic judgment and intra-operative decision making can be discussed during residency. Efforts can also be taken with artificial intelligence to correlate anatomic changes with three-dimensional soft tissue contours within rhinoplasty.

## Declarations

### Human or Animal Participants

This article does not contain any studies with human participants or animals performed by any of the authors.
